# *In vitro* synergistic effect of fluoroquinolone analogues in combination with artemisinin against *Plasmodium falciparum*; their antiplasmodial action in rodent malaria model

**DOI:** 10.1186/s12936-015-0561-2

**Published:** 2015-02-05

**Authors:** Drishti Agarwal, Manish Sharma, Sandeep K Dixit, Roshan K Dutta, Ashok K Singh, Rinkoo D Gupta, Satish K Awasthi

**Affiliations:** Chemical Biology Laboratory, Department of Chemistry, University of Delhi, Delhi, 110007 India; Department of Zoology, University of Delhi, Delhi, 110007 India; Faculty of Life Sciences and Biotechnology, South Asian University, New Delhi, 110021 India

**Keywords:** Fluoroquinolone derivatives, artemisinin, isobologram, antiplasmodial activity

## Abstract

**Background:**

Emergence of drug-resistant parasite strains has surfaced as a major obstacle in attempts to ameliorate malaria. Current treatment regimen of malaria relies on the concept of artemisinin-based combination therapy (ACT).

**Methods:**

Fluoroquinolone analogues, compounds **10**, **12** and **18** were investigated for their anti-malarial interaction in combination with artemisinin *in vitro,* against *Plasmodium falciparum* 3D7 strain, employing fixed-ratio combination isobologram method. In addition, the efficacy of these compounds was evaluated intraperitoneally in BALB/c mice infected with chloroquine-resistant *Plasmodium berghei* ANKA strain in the Peters’ four-day suppressive test.

**Results:**

Promising results were obtained in the form of synergistic or additive interactions. Compounds **10** and **12** were found to have highly synergistic interactions with artemisinin. Antiplasmodial effect was further verified by the convincing ED_50_ values of these compounds, which ranged between 2.31 and 3.09 (mg/kg BW).

**Conclusions:**

*In vivo* studies substantiated the potential of the fluoroquinolone derivatives to be developed as synergistic partners for anti-malarial drug combinations.

## Background

Drug-resistant malaria has emerged as the most undefiable obstacle in the battle against this deadly disease [1,2]. Artemisinin and its analogues, once regarded as the most powerful drugs that cure chloroquine-resistant *Plasmodium falciparum* infections, have also fallen to resistance [3-6]. Therefore, the need of the hour is to ward off the deployment of artemisinin and its analogues as monotherapy, to support WHO’s resolution of advocating artemisinin-based combination therapy (ACT), and ensure their methodical and practicable implementation in all afflicted areas. As the available ACT is only a handful, there is tremendous possibility of the selection of *Plasmodium* strains with acquired resistance towards them. Therefore, the current focus should be directed towards devising alternative ACT. The underlying mechanism behind the therapeutic effect of artemisinin-based combinations is that the artemisinin component rapidly and effectively wipes out most of the parasites, while those that remain are successively annihilated by a high concentration of the partner drug [7]. The efficacy and short half life (< one hour) of the artemisinin component confers protection against development of drug resistance. The long half life companion drug is required to ensure no parasite is left unperturbed. In this manner, the probability that mutant parasites survive and emerge after co-administration of these two drugs is very low.

In spite of the availability of several potent drugs as partners in ACT, quinolones are one of the cardinal classes as they can target both the blood and liver parasite stages [8]. The current status of quinolones as anti-malarials can be traced back to 1962 when Lesher *et al.* [9] discovered nalidixic acid as a by-product of the synthesis of the anti-malarial drug, chloroquine. This discovery paved the way for further development of a vast array of quinolone compounds, along with those in clinical use [10,11]. Presently, primaquine and atovaquone are the only anti-malarials available commercially that target liver stage parasites as well [12,13]. The first quinolone identified to possess activity against multiple parasite forms was endochin, 4(1H)-quinolone compound, in avian malaria models [14]. A long time after its discovery back in the 1940s, other fluoroquinolones, such as norfloxacin, ciprofloxacin, pefloxacin, grepafloxacin, trovafloxacin, enoxacin, and clinofloxacin were evaluated against the malaria parasite *in vitro* [9,15,16] and *in vivo* [17-19]. Although these common antibiotics were found efficacious against both chloroquine-sensitive and -resistant parasites, highly effective concentrations and prolonged treatment regimen (14 days) have restricted their use as sole therapy. These findings support further screening of newer fluoroquinolone compounds as partner drugs.

**Figure 1:**
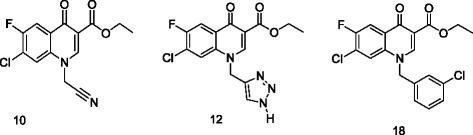
**Chemical structures of compounds 10, 12 and 18.**

The synthesis of two series of fluoroquinolone analogues has been reported previously, amongst which several compounds exhibited significant anti-malarial activity, with very low to negligible toxicity [20]. These are substituted fluoroquinolones with normal and branched chain alkyl groups as well as some polar groups such as -OH, −CN and -C ≡ CH etc. In the present study, three most active compounds (Figure 1) from the aforementioned series were selected, which yielded least inhibitory concentrations, i.e., inhibited the parasite multiplication rate to 50% (IC_50_) at concentrations of < 3 μg/ml (2.56 ± 0.30, 1.33 ± 0.67 and 2.73 ± 0.23 μg/ml ± SE (equivalent to 8.69, 3.79 and 6.93 μM), and exhibited in vitro host cell cytotoxicity IC_50_ values of 142.81, 171.37 and 129.24 μM for compounds **10**, **12** and **18**, respectively. These values are strikingly lower than that of the fluoroquinolones in clinical use. While the reported IC_50_ values (μM) of ciprofloxacin, clinafloxacin, and norfloxacin against 3D7 strain of *P. falciparum in vitro* are 27.77, 37.45 and 53.86, respectively, those of enoxacin and ofloxacin rise up to 121.13 and 152.10. Therefore, it was considered interesting to investigate the fixed-ratio combinatorial interactions of each of these three novel fluoroquinolone derivatives with artemisinin, for treating the erythrocytic stages of *P. falciparum* strain 3D7. A modified isobologram method [21] was followed to assess the synergistic, antagonistic or additive interactions of the combinations. Additionally, on account of their convincing antiplasmodial activity under *in vitro* conditions, it was imperative to assess their efficacy *in vivo*, employing a rodent malaria model.

## Methods

### Antiplasmodial interaction assay of artemisinin and fluoroquinolone analogue combinations

#### Parasite culture

Stock culture of malaria parasite *P. falciparum* 3D7 strain (chloroquine sensitive) was continuously maintained *in vitro* using a CO_2_ incubator under low-oxygen concentration (3%) and high carbon dioxide atmosphere (4%) along with nitrogen (93%), incubated at a temperature of 37°C. The parasites were maintained on O^+^ human red blood cells suspended in a complete culture medium. Each litre of RPMI-1640 aqueous culture medium was prepared with 10.4 g of powdered RPMI-1640 (with glutamine but without sodium bicarbonate), 5.94 g of HEPES buffer, 1 g of dextrose and 40 mg of gentamicin. Complete medium was constituted just before use by adding sterile 5% sodium bicarbonate at the rate of 4 ml per 96 ml, and supplemented with 10% (v/v) pooled O^+^ human serum. Infected erythrocytes were suspended in this culture media initiated at a haematocrit value of 5% and parasitemia was kept between 2 and 4% with sub-culturing done beyond 5%. Medium was changed once a day and percentage parasitemia was monitored using Giemsa stained slides.

### Stock solution of compounds

Artemisinin (Sigma Aldrich, USA) was prepared in DMSO to get the stock solution of 1 mg/ml strength. Compounds **10**, **12** and **18** were synthesized according to the procedure described by Dixit *et al.* [20] and each compound was made to strength of 1 mg/ml stock solution in DMSO. The stock solutions were diluted on the day of experiment to get the desired concentrations for each compound. The highest amount of DMSO in diluted concentrations was 0.125%, and had no effect on parasite growth.

### Preparation of fixed-ratio combinations

In each combination assay, two compounds (Compound A, artemisinin and Compound B, a fluoroquinolone derivative or norfloxacin) were combined in four fixed ratios (4:1, 3:2, 2:3, and 1:4). Approximately eight-fold IC_50_ compound concentration of the respective compound A or B was taken as 100% (calculated to be 32 nM, 69.50 μM, 30.34 μM, 55.40 μM, and 430.88 μM for artemisinin, compound **10**, compound **12,** compound **18** and norfloxacin, respectively), so that IC_50_ of the individual compound falls in between third and fourth two-fold serial dilution.

### Plate preparation for antiplasmodial interaction assay

Compound dilutions of each combination solution were made in sterile, flat-bottomed, 96-well tissue culture plates as described by Fivelman *et al*. [21]. Six times two-fold serial dilution was done for each combination in triplicate. Each well contained a total volume of 200 μl of complete culture medium with or without compound and pre-synchronized infected red blood cells (1% parasitemia at 2.5% haematocrit). Control cultures (without compound) were maintained on the same plate in triplicate. Two 96-well plates (for six combinations) were used for each combination experiment. The plates were stacked in a CO_2_ incubator and incubated at 37°C for 48 hours.

### Slide preparation, staining and assessment

After 48-hour incubation, thin blood smear slides were prepared, air dried, methanol fixed, and stained in Giemsa solution for 40 min. After staining, slides were removed from coupling jar, washed in running tap water and air dried. The Giemsa-stained slides were examined for counting the number of parasites in random adjacent microscopic fields, equivalent to about 4,000 erythrocytes at 1,000 × magnification. Per cent parasitemia was calculated. Reproducibility of counts was checked by two other readers to maintain the quality control.

### Isobologram preparation and data analysis

For each combination assay, IC_50_ was calculated from two sets of concentration response graphs, each containing compound alone curve and four combination curves.

The sum FIC of each combination ratio of two combined compounds shows that the drug-drug schizontocidal interaction between them [22] was determined by the following equation:

$$ \mathrm{Sum}\ \mathrm{F}\mathrm{I}\mathrm{C}\ \left(\boldsymbol{\sum}\mathrm{F}\mathrm{I}\mathrm{C}\right)=\frac{\mathrm{IC}50\ \mathrm{of}\ \mathrm{A}\ \mathrm{in}\ \mathrm{mixture}}{\mathrm{IC}50\ \mathrm{of}\ \mathrm{A}\ \mathrm{alone}}+\frac{\mathrm{IC}50\ \mathrm{of}\ \mathrm{B}\ \mathrm{in}\ \mathrm{mixture}}{\mathrm{IC}50\ \mathrm{of}\ \mathrm{B}\ \mathrm{alone}} $$

***Ʃ***FIC <1 represents synergism, ***Ʃ***FIC > = 1 and <2 represents additive interaction, ***Ʃ***FIC > = 2 and <4 represents slight antagonism while ***Ʃ***FIC > = 4 represents marked antagonism [23-25]. Mean FICs of the combinations were compared by frequency distribution using GraphPad Prism 5, to define if a compound was superior to the other one, when combined with artemisinin.

### *In vivo* efficacy of fluoroquinolone analogues using rodent malaria model

Evaluation of the curative potential of the fluoroquinolone derivatives was done using the method described by Ryley and Peters, 1970 (rodent malaria four-day suppressive test; Peters’ four-day suppressive test) [26,27]. Rodent malaria parasite *Plasmodium berghei* ANKA was used.

### Experimental animals

Immuno-compromised BALB/c inbred albino mice (25–30 g) of the male sex were obtained from the Animal Facility Centre of the Department of Zoology, University of Delhi. The animals were fed *ad libitum* with standard feed and had free access to water. They were maintained under standard conditions of humidity, temperature (25°C) and 12 hours light/darkness cycles. The animals were acclimatized for two weeks before the commencement of the study and were ensured to exclude all zoonotic agents.

### Test procedure

Day 0: Heparinized blood was withdrawn from an infected donor mouse with approximately 25-30% parasitemia, and diluted in 1x PBS to 10^8^ parasitized erythrocytes per mL. An aliquot of 0.2 mL (=2 × 10^7^ parasitized erythrocytes) of this suspension was injected intraperitoneally (ip) into experimental groups of five mice each. One to three hours post-infection, the experimental groups were treated with varying doses of each of the test compounds (0.5, 1, 10, 25 mg/kg BW) by the ip route. Each compound was made to strength of 5 mg/ml stock solution in 10% DMSO and administered according to desired concentration and individual body weight. Artemisinin was given to the standard drug group and 0.2 mL of normal saline to the negative control group.

Day 1, 2 and 3: 24 hours, 48 hours and 72 hours post-infection, the experimental groups of mice were treated again with the same dose and by the same route as on day 0.

Day 4: 24 hours after the last treatment (i.e., 96 hours post-infection), blood was drawn from the tail region of mice and smears were prepared. These were stained with Giemsa for microscopic analysis by counting four fields of approximately 500 erythrocytes per slide, for five replicates of each sample, to determine the parasitemia percentage and hence assess the anti-malarial efficacy of the test compounds. Differences in parasitemia percentage between treated groups and untreated animals were analysed by a one-way ANOVA test using IBM SPSS Statistics 16.0 and differences considered significant if P < 0.05. Furthermore, the difference between the mean value of the negative control group (taken as 100%) and those of the experimental groups was calculated and expressed as per cent inhibition (= activity) using the equation below and hence ED_50_ value was calculated graphically.

$$ \mathrm{Per}\ \mathrm{cent}\ \mathrm{inhibition}\ \left(\mathrm{activity}\right) = 100\ \hbox{--}\ \left(\mathrm{Mean}\ \mathrm{parasitemia}/\mathrm{control}\right) \times 100 $$

Untreated control mice typically died approximately one week after infection. Treated mice were observed for a period of 30 days, and the survival time (in days) was recorded. The mean survival time was calculated in comparison to untreated (Normal saline) and standard drug (artemisinin) treated groups. Differences in survival time between treated groups and untreated animals were analysed by Log-rank (Mantel-Cox) test using GraphPad Prism 5 and differences considered significant if P <0.005. Observations concerning adverse effects due to the compounds were recorded.

### LD_50_ Test

LD_50_ test was carried out on BALB/c mice using different dosages of various compounds: 50, 100, 200, 500, 600, 800 and 1000 mg/kg BW ip and the animals were observed for 7 days. Therapeutic Index (TI) values were determined by the formula:

$$ \mathrm{Therapeutic}\ \mathrm{I}\mathrm{ndex}\left(\mathrm{T}\mathrm{I}\right) = \frac{\mathrm{Median}\ \mathrm{lethal}\ \mathrm{dose}\ \left({\mathrm{LD}}_{50}\right)}{\mathrm{Median}\ \mathrm{effective}\ \mathrm{dose}\ \left({\mathrm{ED}}_{50}\right)} $$

## Results

### Antiplasmodial interactions between artemisinin and fluoroquinolone analogues

**Figure 2:**
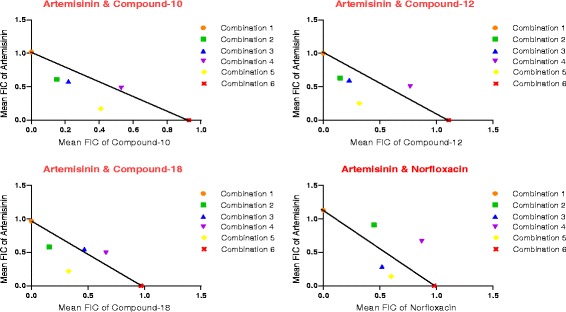
**Isobolograms showing interaction between artemisinin and fluoroquinolone derivatives; artemisinin and norfloxacin against** ***Plasmodium falciparum*** **3D7 strain.**

Various substituted fluoroquinolones were synthesized by the earlier reported procedure [20]. The pharmacophore of fluoroquinolones shows striking similarity with chloroquine, which has been the forerunner for malaria treatment for the past 50 years [28]. Both fluoroquinolones and choloroquine contain chlorine at position 7. A vast array of fluoroquinolones have been investigated and henceforth proven effective against *P. falciparum*. Here, three substituted fluoroquinolone compounds, **10**, **12** and **18** were chosen from previous *in vitro* study [20], and were tested in combination with artemisinin *in vitro* against *P. falciparum* chloroquine-sensitive 3D7 strain, using norfloxacin as the standard drug. These compounds were found to have synergistic and additive drug-drug interactions. In every combination assay IC_50_ was determined from two sets of drug response curves obtained from each replicate, each set representing four combination curves and a curve of drug/compound alone. Mean FIC_50_ values derived from these curves are tabulated in Table 1, for each fluoroquinolone derivative combination with artemisinin, and combination of norfloxacin with artemisinin. Sum of FICs are presented in isobolograms (Figure 2). The isobolograms show that anti-malarial interaction of the fluoroquinolone derivatives *in vitro* with artemisinin is not antagonistic. Compound **10** in combination with artemisinin shows synergistic antiplasmodial interaction in three of the four fixed-ratio combinations evaluated and additive in the remaining one. Similarly the combination of compound **12** and artemisinin displays synergistic interaction in three combinations and additive in one. Interaction of artemisinin with compound **18** was found to be synergistic in two combinations, while additive in the other two. The standard drug used in the study, norfloxacin, when combined with artemisinin shows synergistic interaction in two combinations, while additive in the remaining two. Combination of compound 10 with artemisinin tended most towards synergism, with respect to all the other compounds (Mean ƩFIC ± SD = 0.788 ± 0.177), as observed by frequency distribution. Combinations of all fluoroquinolone analogues were superior to that of norfloxacin (Mean ƩFIC ± SD = 1.11 ± 0.394), which tended slightly towards antagonism.

**Table 1:** **Interaction between artemisinin and various fluoroquinolones analogs (compounds 10, 12 and 18) against** ***Plasmodium falciparum*** **(3D7 strain) at six different preparations**

**Combination solution (Ratio A:B)**	**Drug A (Artemisinin) Mean FIC**_**50**_ **± SE** ^**a**^	**Drug B (Compound-10) Mean FIC**_**50**_ **± SE** ^**a**^	**Σ FICs, interaction** ^**b**^
1 (5:0)	1.02 ± 0.04	0	
2 (4:1)	0.61 ± 0.01	0.15 ± 0.02	0.76 SYN
3 (3:2)	0.58 ± 0.03	0.22 ± 0.02	0.80 SYN
4 (2:3)	0.48 ± 0.02	0.53 ± 0.03	1.01 ADD
5 (1:4)	0.17 ± 0.03	0.41 ± 0.03	0.58 SYN
6 (0:5)	0	0.93 ± 0.04	
**Combination solution (Ratio A:B)**	**Drug A (Artemisinin) Mean FIC**_**50**_ **± SE** ^**a**^	**Drug B (Compound-12) Mean FIC**_**50**_ **± SE** ^**a**^	**Σ FICs, interaction** ^**b**^
1 (5:0)	1.0 ± 0.02	0	
2 (4:1)	0.63 ± 0.03	0.15 ± 0.003	0.78 SYN
3 (3:2)	0.60 ± 0.03	0.23 ± 0.02	0.83 SYN
4 (2:3)	0.50 ± 0.02	0.77 ± 0.03	1.27 ADD
5 (1:4)	0.25 ± 0.004	0.32 ± 0.01	0.56 SYN
6 (0:5)	0	1.11 ± 0.08	
**Combination solution (Ratio A:B)**	**Drug A (Artemisinin) Mean FIC**_**50**_ **± SE** ^**a**^	**Drug B (Compound 18) Mean FIC**_**50**_ **± SE** ^**a**^	**Σ FICs, interaction** ^**b**^
1 (5:0)	0.97 ± 0.07	0	
2 ( 4:1)	0.58 ± 0.02	0.16 ± 0.01	0.73 SYN
3 (3:2)	0.55 ± 0.03	0.47 ± 0.03	1.02 ADD
4(2:3)	0.49 ± 0.04	0.66 ± 0.03	1.15 ADD
5 (1:4)	0.22 ± 0.01	0.33 ± 0.02	0.55 SYN
6 (0:5)	0	0.97 ± 0.08	
**Combination solution (Ratio A:B)**	**Drug A (Artemisinin) Mean FIC**_**50**_ **± SE** ^**a**^	**Drug B (Norfloxacin) Mean FIC**_**50**_ **± SE** ^**a**^	**Σ FICs, interaction** ^**b**^
1 (5:0)	1.13 ± 0.06	0	
2 (4:1)	0.91 ± 0.03	0.45 ± 0.02	1.36 ADD
3 (3:2)	0.29 ± 0.02	0.52 ± 0.04	0.81 SYN
4 (2:3)	0.66 ± 0.02	0.87 ± 0.03	1.53 ADD
5 (1:4)	0.14 ± 0.01	0.60 ± 0.02	0.74 SYN
6 (0:5)	0	0.98 ± 0.04	

^a^Standard error (n = 3); ^b^ADD, additive; SYN, synergistic.

In principle, components of anti-malarial combinations should target different metabolic pathways. This condition is being theoretically met in the combinations being evaluated. The hypothesized mechanisms of action of artemisinin include haem alkylation, inhibition of PfATP6 (SERCA-type enzyme), parasite membrane damage [29-31]. On the contrary, fluoroquinolones are the only class of antimicrobial agents that are direct inhibitors of bacterial DNA synthesis. They inhibit two bacterial enzymes: DNA gyrase, particularly the A subunit, and topoisomerase IV, which have essential roles in DNA replication [32]. *Plasmodium falciparum* contains a functional apicoplast, an organelle of prokaryotic origin. The 27–35 kb circular genome of apicoplast requires bacterial type DNA gyrase for its duplication [33-35]. This is the most likely explanation for inhibitory activity of fluoroquinolones against the parasite, which is being enhanced on interaction with artemisinin either synergistically or additively, analysed using fixed-ratio isobolograms. However, the exact mode of action of fluoroquinolones against malaria parasites is still ambiguous.

### Antiplasmodial activity of synthetic fluoroquinolones against *Plasmodium berghei in vivo*

**Figure 3:**
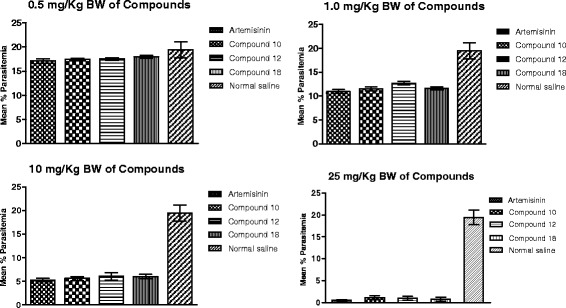
**Effects of various compounds and artemisinin on established** ***P. berghei*** **infections in mice.** The experimental hosts were infected on day 0 and treated intraperitoneally with normal saline; compounds 10, 12, 18 and artemisinin at 0.5, 1.0, 10, or 25 mg · Kg^−1^ BW · day^−1^ on days 0 to 3, as described by Ryley and Peters. Data expressed as Mean ± SD of five mice per condition.

**Figure 4:**
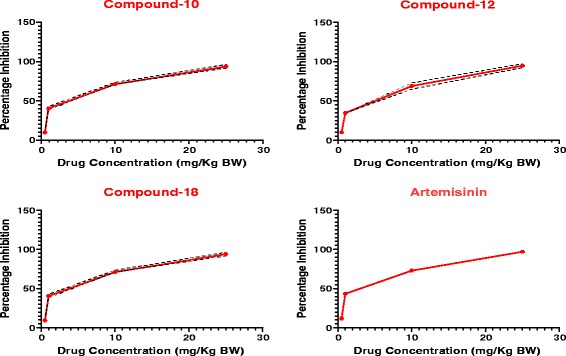
**Dose–response curves of fluoroquinolone derivatives and artemisinin, against** ***in vivo*** **blood stages of** ***Plasmodium berghei*** **strain ANKA.** Data expressed as Mean ± SD of five mice/compound.

**Figure 5:**
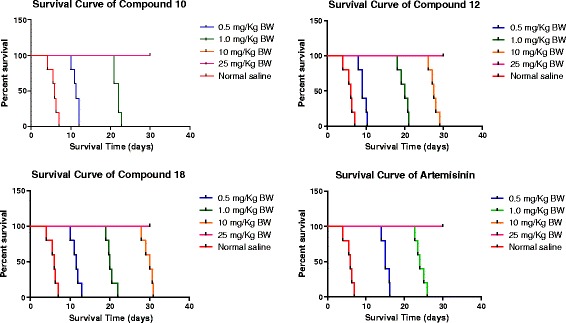
**Kaplan Meier survival analysis curves of BALB/c mice, administered drugs once daily ip for four consecutive days (5 mice per group).** Results between test and control were significant by P < 0.005 as analysed by Log-rank test.

It was observed that there was a reduction in the levels of parasitemia in all the test groups, as well as that of the standard drug (artemisinin) group. However, the reverse was the case for the negative control group, as there was a marked increase in parasitemia level. The *in vivo* anti-malarial activity of the various test compounds, after conducting Peters’ four-day suppressive test, is presented in Figure 3. Results were significant as analysed by ANOVA (P <0.05). The ED_50_ values were calculated to be 2.31, 3.09, 2.60, and 1.72 mg/kg BW for each of the compounds **10**, **12**, **18**, and artemisinin, respectively, as indicated in Table 2, and represented graphically in Figure 4. The mean survival time (MST) values of the treated groups were significantly higher than that of control and were comparable to that of the standard drug, artemisinin (Figure 5). The mice treated with varying doses of each of the fluoroquinolone derivatives survived beyond one week, but 0.5 mg/kg BW treated mice died nine to 12 days post treatment. Artemisinin-treated mice on the other hand, survived beyond two weeks in all the groups.

**Table 2:** **Antiplasmodial activity of fluoroquinolone derivatives against** ***Plasmodium berghei*** **strain ANKA**

**Compound**	**Mean ED**_**50**_ **(mg/kg BW) ± SE** ^**a**^
10	2.31 ± 0.19
12	3.09 ± 0.22
18	2.60 ± 0.18
Artemisinin	1.72 ± 0.15

^a^Standard error (n = 5).

The results show that two out of the three compounds (**10** and **18**) exhibited appreciable antiplasmodial activity, reflected by their ED_50_ values, comparable to that of the standard drug, artemisinin. The study on MST demonstrated a dose-dependent increase in the number of days the mice survived in various groups survived post-four-day treatment. MST values for compound **10** (with lowest ED_50_ = 2.31 mg/kg BW) were the most proximal to those obtained for artemisinin, while the next to follow was compound **18** (ED_50_ = 2.60 mg/kg BW). All the results were significant (P < 0.005) as analysed by Log-rank (Mantel-Cox) test. Therefore, they serve as promising candidates for further research. No significant adverse side effects, i.e., physical signs such as gasping for air, loss of appetite, feeling sleepy, or weight loss were observed in compound-treated groups, even at the highest dose administered, indicating that the compounds are well tolerated by the biological system and may be toxic at doses much higher than those required for their therapeutic effects. Thus, compounds **10** and **18** could be excellent candidates for combination therapy.

### LD50 Test

BALB/c mice died at 1000 mg/kg BW of all the compounds and could tolerate 500 mg/kg BW. However, at 800 mg/kg BW, half the population of mice died. Therapeutic indices were determined as 346.32, 258.90, 307.69 for compounds **10**, **12** and **18**, respectively.

## Conclusions

Substantial evidence has been furnished that the fluoroquinolone analogues under assessment show inhibitory activities against the blood stages of the malaria parasite, with ED_50_ values in single-digit, micro-molar range. The above analogues have been shown to display a synergistic mode of interaction with artemisinin, in majority of fixed-ratio combinations analysed *in vitro*. The results of this work further justify the use of novel, synthetic fluoroquinolones in the treatment of malarial infection. It is therefore concluded that compounds **10**, **12** and **18** have appreciable anti-malarial activity that can be exploited for the production of modern anti-malarial pharmaceuticals. Drugs that target both the liver and blood stages of malaria are urgently required to reduce the disease's extensive worldwide morbidity and mortality [33]. Hence, it would be highly intriguing to evaluate the possibility of these compounds to target the liver stage parasites and dormant hypnozoites as well, which would strengthen their position as potential, all-purpose anti-malarial drug candidates. To find a lead molecule for drug development, extensive SAR is needed and further investigation in this direction is under progress.

## Abbreviations

ACT: Artemisinin-based combined therapy
RPMI: Roswell Park Memorial Institute (cell culture medium)
HEPES: 4-(2-hydroxyethyl)-1-piperazine-ethanesulfonic acid
IC_50_: Median inhibition concentration of growth
ED_50_: Median effective dose
FIC: Fractional inhibitory concentration
DMSO: Dimethyl sulphoxide, MST, mean survival time
SAR: Structural-activity relationship
BW: Body weight
